# Identification of novel lactate metabolism-related lncRNAs with prognostic value for bladder cancer

**DOI:** 10.3389/fphar.2023.1215296

**Published:** 2023-09-13

**Authors:** Xiushen Wang, Jing Pan, Qiutong Guan, Ninghui Ren, Ping Wang, Minjie Wei, Zhenhua Li

**Affiliations:** ^1^ Department of Urology, The Fourth Affiliated Hospital, China Medical University, Shenyang, China; ^2^ College of Pharmacy, China Medical University, Shenyang, China; ^3^ School of Life Science and Biopharmaceutics, Shenyang Pharmaceutical University, Shenyang, China

**Keywords:** bladder cancer, lncRNAs, molecular subtype, prognostic model, lactate metabolism

## Abstract

**Background:** Bladder cancer (BCA) has high recurrence and metastasis rates, and current treatment options show limited efficacy and significant adverse effects. It is crucial to find diagnostic markers and therapeutic targets with clinical value. This study aimed to identify lactate metabolism-related lncRNAs (LM_lncRNAs) to establish a model for evaluating bladder cancer prognosis.

**Method:** A risk model consisting of lactate metabolism-related lncRNAs was developed to forecast bladder cancer patient prognosis using The Cancer Genome Atlas (TCGA) database. *Kaplan‒Meier* survival analysis, receiver operating characteristic curve (ROC) analysis and decision curve analysis (DCA) were used to evaluate the reliability of risk grouping for predictive analysis of bladder cancer patients. The results were also validated in the validation set. Chemotherapeutic agents sensitive to lactate metabolism were assessed using the Genomics of Drug Sensitivity in Cancer (GDSC) database.

**Results:** As an independent prognostic factor for patients, lactate metabolism-related lncRNAs can be used as a nomogram chart that predicts overall survival time (OS). There were significant differences in survival rates between the high-risk and low-risk groups based on the *Kaplan‒Meier* survival curve. decision curve analysis and receiver operating characteristic curve analysis confirmed its good predictive capacity. As a result, 22 chemotherapeutic agents were predicted to positively affect the high-risk group.

**Conclusion:** An lactate metabolism-related lncRNA prediction model was proposed to predict the prognosis for patients with bladder cancer and chemotherapeutic drug sensitivity in high-risk groups, which provided a new idea for the prognostic evaluation of the clinical treatment of bladder cancer.

## 1 Introduction

Globally, bladder cancer (BCA) is the most common urological malignancy and requires lifelong monitoring after diagnosis (Dobruch and Oszczudłowski, 2021). Twenty to thirty percent of BCA patients have progressed to muscle-invasive BCA (MIBCA) when diagnosed (Fletcher et al., 2011). Nearly 50% of MIBCA patients develop tumor metastasis after radical cystectomy (RC). BCA is estimated to cause 356,000 new cases and 145,000 deaths yearly (Antoni et al., 2017). Consequently, the guidelines recommend treating MIBC with neoadjuvant chemotherapy (NAC) and RC (Milowsky et al., 2016). Approximately 50% of MIBCA patients cannot tolerate suppressive adverse events resulting from chemotherapy, leading to treatment delays in nonresponders (Hanna et al., 2018). To improve cancer patients’ clinical efficacy and prognosis, clarifying BCA pathogenesis and determining targets for diagnosis and treatment are imperative.

Urothelial BCA (UBCA) is one of the earliest cancers considered to have immunogenicity. With the FDA’s approval of immune checkpoint inhibitors (ICIs) and pan-FGFR inhibitors, PD-1/PD-L1 therapy has shown an impressive lasting response in UBCA patients. However, the response rate has been low (Eckstein et al., 2019). To maintain uncontrolled growth and proliferation, BCA may use aerobic glycolysis-dependent metabolism (the Warburg effect) as the primary energy source (Yang et al., 2011). High lactic acid levels and subsequent acidification caused by glycolytic metabolic transformation may promote carcinogenesis and contribute to invasion, immune escape, metastasis, and chemoradiotherapy resistance (Wang et al., 2020). In addition, the Warburg effect is a feature of MIBCA and nonmuscle invasive BCA (NMIBC) (Burns et al., 2021). A significant portion of the glucose storage is converted into lactic acid by lactate dehydrogenase-A (LDH-A), resulting in glucose being used to promote growth, regardless of oxygen levels (Kim et al., 2006). *In vitro*, overexpression of LDH-A promoted BCA proliferation, invasion, and migration by stimulating epithelial-mesenchymal transformation (EMT) (Jiang et al., 2016). The metabolic state of tumor cells influences their interactions with the tumor microenvironment (TME), which is crucial for antitumor immunity (Bader et al., 2020). As lactic acid levels increase in the TME, tumor-associated macrophages differentiate into M2 subtypes, while activated macrophages promote tumor invasion through the CCL17/CCR-4/Mtorc1 signaling axis (Zhang et al., 2021). Lactic acid derived from tumor cells induces GPR81 expression in dendritic cells through a paracrine mechanism, inhibiting immune cell antigen presentation (Brown et al., 2020). These reports suggest that an in-depth understanding of lactate metabolism in BCA will provide new opportunities to predict the disease life cycle and find targets for tumor immunotherapy.

A long noncoding RNA (lncRNA) is an RNA transcribed over 200 nucleotides without the capability to code for proteins. Various cancers, including BCA, can be initiated and progress at different levels, including epigenetic, transcriptional, and posttranscriptional regulation (Iyer et al., 2015). In BCA patients, overexpression of Aly/REF export factor (ALYREF) promotes cell proliferation through PKM2-mediated glycolysis and high expression of pyruvate kinase M2 (PKM2), and ALYREF predicts poor survival (Wang et al., 2021). The low expression of AlkB homolog 5 (ALKBH5) results in poor prognosis in BCA patients, inhibits progression in a m6A-dependent manner, and sensitizes BCA cells to cisplatin through the casein kinase 2 (CK2)α-mediated glycolytic pathway (Yu et al., 2020). Due to their regulatory influences on BCA metabolism, lncRNAs are considered potential targets for drug screening and are a promising area of research.

In recent years, using high-throughput sequencing and data analysis in biomedical research has become increasingly important in identifying biomarkers, predicting prognosis, and monitoring recurrence and stratification (Zhang et al., 2019). Many studies have used a variety of biomarkers to establish clinical patient diagnosis or prognosis prediction models (Liu et al., 2021). Many studies have focused on hypoxia modulating tumor immune responses, while lactate has mainly been ignored in BCA metabolism.

Herein, LM_lncRNAs were analyzed using bioinformatics, a prognostic model for BCA was established, chemotherapy-targeted drugs were explored based on lactate metabolism groups, and a prediction model was developed for the prognosis of BCA. This study may benefit the innovation of customized precision diagnosis and treatment strategies for BCA.

## 2 Methods

### 2.1 Data acquisition

TCGA data are freely available to the public, and this study strictly follows access policies and publication guidelines. BCA RNA expression data were downloaded from TCGA GDC’s official website (https://portal.gdc.cancer.gov/). A total of 408 BCA patients were evaluated for gene expression. This study included variables such as the age and sex of the participants, American Joint Committee on Cancer (AJCC) stage, histological grade, and survival rate. We excluded 11 samples of BCA patients with OS< 30 days and one sample without OS recorded. All remaining patients were included in our study. In this study, we included 397 patient samples and 19 paracancerous samples (Table 1). To select mRNAs with a *p*-value less than 0.05, fragments per kilobase million (FPKM) were converted into transcripts per million (TPM). The Molecular Signatures Database (MSigDB) contains a gene set related to lactate (Hallmark-lactate) (Liberzon et al., 2015).

**TABLE 1:** The clinical characteristics of patients in the TCGA dataset

Variable	Number of samples
Gender	
Male/Female	294/103
Age	
≤65/>65	159/238
Stage	
I/II/III/IV/NA	2/124/137/132/2
Grade	
High/Low/UN	376/18/3
T	
T0/T1/T2/T3/T4/UN	1/3/114/190/58/31
M	
M0/M1/MX/UN	187/10/198/2
N	
N0/N1/N2/N3/NX/UN	229/45/76/7/36/4

### 2.2 Identification of differentially expressed LM_lncRNA

Our screening procedure used a |log_2_FC| > 1 and a false discovery rate (FDR) < 0.05. The limma package was also used to identify all differentially expressed lncRNAs (Ritchie et al., 2015). It was determined whether there was a relationship between the LM_mRNAs in the sample and all lncRNAs differentially expressed data calculated by Pearson correlation. A correlation was demonstrated if |*R* ^2^| > 0.3 and *p* < 0.001.

### 2.3 Development of the LM_lncRNA prognostic signature

Based on univariate Cox analysis, lncRNAs predict overall survival (OS) in BCA patients. Afterward, we selected lncRNAs with independent prognostic characteristics using multivariate Cox regression. In this study, we selected lncRNAs that are independent prognostic factors for patient survival using the survminer software package. The regression coefficient of the multivariate Cox regression model was multiplied by the linear combination of expression levels to generate a prognostic risk score based on LM_lncRNAs.

This model can be expressed as follows:

$$\left.\text{Risk score}=@\text{Expr of lncRNA }\left(1\right)\times \text{coefficient of lncRNA }\left(1\right)]+@\text{Expr of lncRNA }\left(2\right)\times \text{coefficient of lncRNA }\left(2\right)]+\ldots \ldots +@\text{Expr of lncRNA }\left(n\right)\times \text{coefficient of lncRNA }\left(n\right)\right]$$

$$Riskscore=\sum_{i=0}^{N}\left(\text{Expi}*\beta i\right)$$

In this formula, Expi is the expression level of each prognostic lncRNA, and the coefficient is βi. Furthermore, patients were divided into high-risk and low-risk groups based on the median lactate-related risk scores calculated by the formula above. *Kaplan‒Meier* survival curves, receiver operating characteristic curves (ROCs), and C-indices were used to predict patient outcomes and decision curve analysis (DCA).

### 2.4 Signature validation of LM_lncRNA

The TCGA dataset (dataset 1) contains 393 patients divided into two subgroups based on random selection. There were 197 patients in validation set 1 and 196 in validation set 2 (dataset 2). TCGA datasets were analyzed, prognostic features were identified, and the model’s performance was validated in 2 datasets, validation sets 1 and 2. Having validated the prognostic value of lncRNA models based on the LM_lncRNA signature, we validated its impact on survival outcomes in BCA patients. The OS effects of prognostic factors were compared between high-risk and low-risk patients using log-rank tests and *Kaplan‒Meier* survival curves. To evaluate the accuracy of the immune profile derived from the survival ROC software package, we calculated the area under the curve (AUC) using time-dependent ROC curves.

### 2.5 Coexpression network construction

Using Cytoscape, we constructed a correlation network between mRNAs and lncRNAs. With the help of the R software package ggalluvial, we analyzed the relationship between lncRNAs and risk.

### 2.6 Predictive nomograms and GSEA enrichment analysis

Separate gene expression analyses were conducted for high- and low-risk groups related to lactate metabolism (Subramanian et al., 2005). With an FDR q-value <0.25, the difference was considered statistically significant. To estimate the OS of patients at 1, 3, and 5 years, we constructed a Norman diagram and calibrated the statistics using the RMS package. After a calibration curve was developed, statistically significant values (*p* < 0.05) were calculated and compared with patient predictions at the 3- and 5-year marks.

### 2.7 Immunity reaction and sensitivity to immunotherapies/chemotherapies

Infiltration of immune cells in tumors in the high-risk and low-risk groups was estimated using the ESTIMATE algorithm (Yoshihara et al., 2013). Identifying immune checkpoints and m6A modification enabled quantification of immune function in high- and low-risk populations.

Every patient with BCA can be predicted to respond to chemotherapy using the Genomics of Cancer Drug Sensitivity database (GDSC) (Yang et al., 2013). The GDSC database predicts chemosensitivity in patients with two types of BCA. A half-maximum inhibitory concentration (IC50) was predicted using ridge regression in the “pRRophetic” package (Geeleher et al., 2014). Ten cross-validations are conducted to calculate accuracy.

### 2.8 Statistical analysis

R software was used for all data analysis and visualization (version 4.1.2). If the distribution of the groups was not expected or the variance was unknown, Wilcoxon rank-sum tests or Kruskal‒Wallis tests were used to compare them. Cox regression analysis was conducted on both the univariate and multivariate data. Survival differences were assessed using log-rank tests. We assessed the sensitivity and specificity of BCA prognosis and other clinicopathological features by calculating ROC curves and C-indices. The statistics were considered significant if *p* < 0.05.

## 3 Results

**FIGURE 1:**
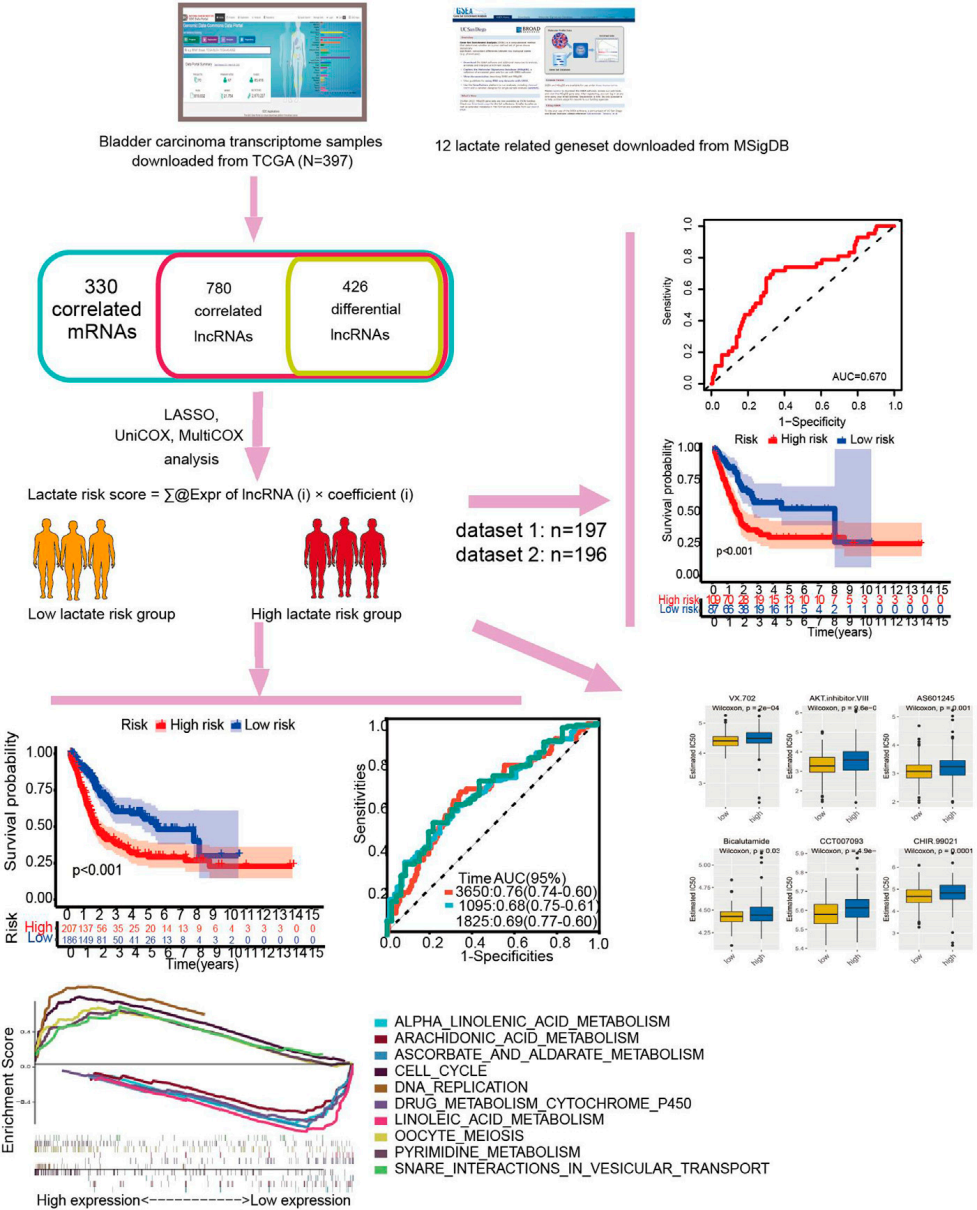
Study flowchart. Three hundred thirty lactate-related mRNAs and 780 related lncRNAs (LRLs) were obtained from the TCGA and MSigDB databases. Then, 426 lactate-related differentially expressed lncRNAs (LDELs) were identified according to their differential expression in the tumor and adjacent tumor. Next, univariate Cox, Lasso, and multivariate Cox analyses were applied to screen for prognostic LDELs. Based on this analysis, a 5-LDEL signature was constructed. Subsequently, GSEA analyses, immune-related analyses, m^6^A-related analyses, and drug sensitivity assays were applied to identify the potential function of this signature. Finally, 2 internal validations were conducted to explore the expression and function of these LDELs.

In Figure 1, a flow chart describes this study in more detail.

### 3.1 Identification of significantly enriched LM_lncRNAs

**FIGURE 2:**
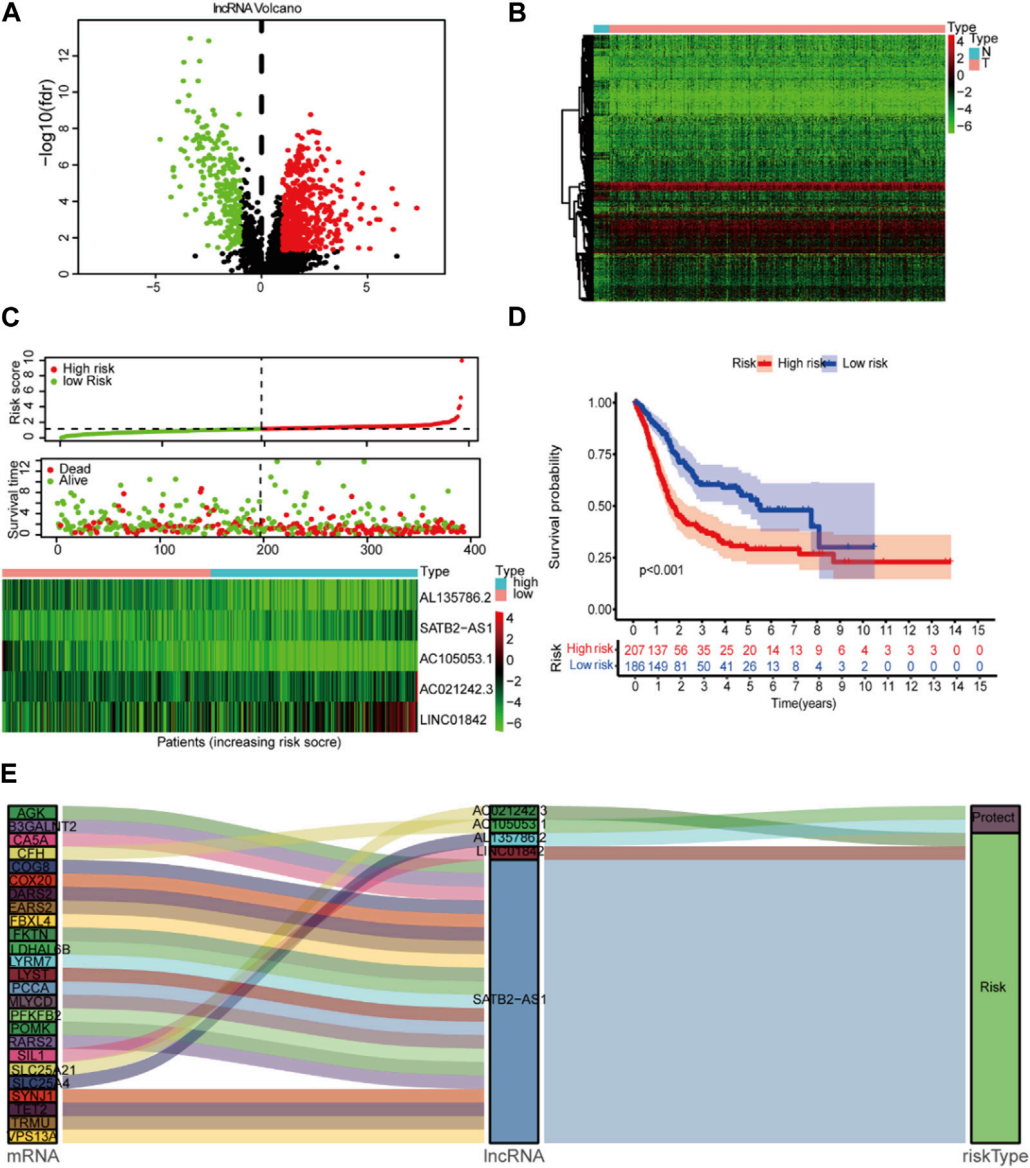
Lactate signature construction. **(A)** Volcano map for differentially expressed lncRNAs. **(B)** Heatmap for differentially expressed lncRNAs. **(C)** Risk score distribution and survival status of the two risk groups. **(D)** *Kaplan‒Meier* curve analysis (*K-M* curve analysis) for the cohort. **(E)** The Sankey diagram presents the detailed connection between lactate-related lncRNAs and lactate-related genes.

Twelve GSEA gene sets were related to lactate metabolism in the MSigDB database, and all of the lncRNAs were extracted, totaling 330. A total of 306 lncRNAs were enriched for lactate metabolism-related pathways after intersection processing with the entire gene set of the sample. Based on the Pearson correlation between mRNAs and lncRNAs in BCA, we screened lncRNAs significantly associated with lactate metabolism. We obtained 780 candidate gene expression data of lncRNAs with the criteria of |*R* ^2^| > 0.3 and *p* < 0.001 (Supplementary Table S2, S3). Among them, 548 lncRNAs were overexpressed, and 232 lncRNAs were downregulated. A total of 426 differential lncRNAs were identified with *p* < 0.05 and |log_2_ FC| > 1 criteria. The heatmap and volcano map of the different analyses are shown in Figures 2A,B.

### 3.2 Construction and multivariate evaluation of the prognostic significance of LM_lncRNA

This study included 397 BCA patients and 306 LM_lncRNAs in the TCGA cohort to determine prognostic risk models. The association between survival and LM_lncRNAs was determined by univariate Cox regression analysis. As a result, when the *p* < 0.05, we found seven lncRNAs significantly associated with OS in BCA patients. Figure 2C shows the prediction model constructed from five lncRNAs as the result of multivariate stepwise Cox regression analysis. A prognostic model based on LM_lncRNA was developed by dividing patients into two categories based on median risk scores. Compared to the low-risk group, the high-risk group had a shorter mortality and survival time (Figure 2D).

A prognostic risk score formula composed of these five lncRNAs is as follows:

$$\text{Risk score}=\left(1.34455\times \text{Expr of SATB}2-\text{AS}1\right)+\left(0.09399\times \text{Expr of AC}021242.3\right)+\left(-6.07924\times \text{Expr of AC}105053.1\right)+\left(-4.23667\times \text{Expr of AL}135786.2\right)+\left(0.19263\times \text{Expr of LINC}01842\right)$$

In Cox regression analysis, three of these LM_lncRNAs, SATB2-AS1, AC021242.3, and LINC01842, showed positive coefficients, suggesting that their high expression is associated with poorer OS. While the coefficients of AC105053.1 and AL135786.2 were negative, the Sankey diagram indicated that this lncRNA was protective (Figure 2E).

**FIGURE 3:**
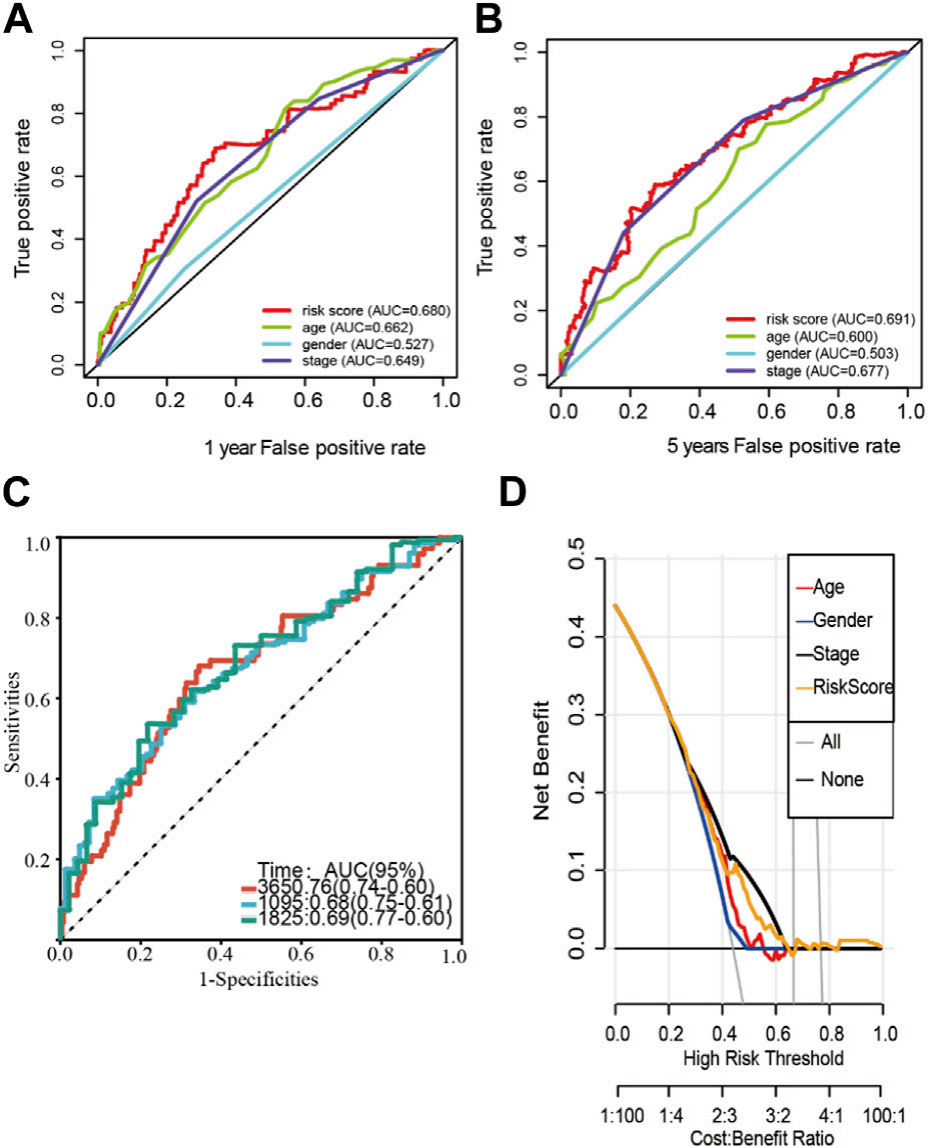
Stability verification of the lactate-related lncRNA signature model in the training cohort. **(A-B)** The 1-year AUC of this signature lncRNA was 0.681, and the 5-year AUC was 0.691. **(C)** The predicted 3-, 5-, and 10-year survival receiver operating characteristic (ROC) curves of the new lncRNA features were 0.67, 0.68, and 0.69, respectively. **(D)** The model’s decision curve analysis (DCA) also shows that the model has good profitability.

An analysis of clinicopathological manifestations and LM_lncRNA prognostic features was conducted using a heatmap. Meanwhile, the 1-year AUC of this signature lncRNA was 0.681, and the 5-year AUC was 0.691, which was superior to standard clinicopathological features in predicting BCA prognosis (Figures 3A,B). Over the 3-, 5-, and 10-year periods, the survival ROCs were 0.67, 0.68, and 0.69, indicating that the predictive ability of the model was still good after 10 years (Figure 3C). As shown by DCA, the model had good profitability based on its C-index of 0.648 (Figure 3D).

### 3.3 Validation of the LM lncRNA signature

**FIGURE 4:**
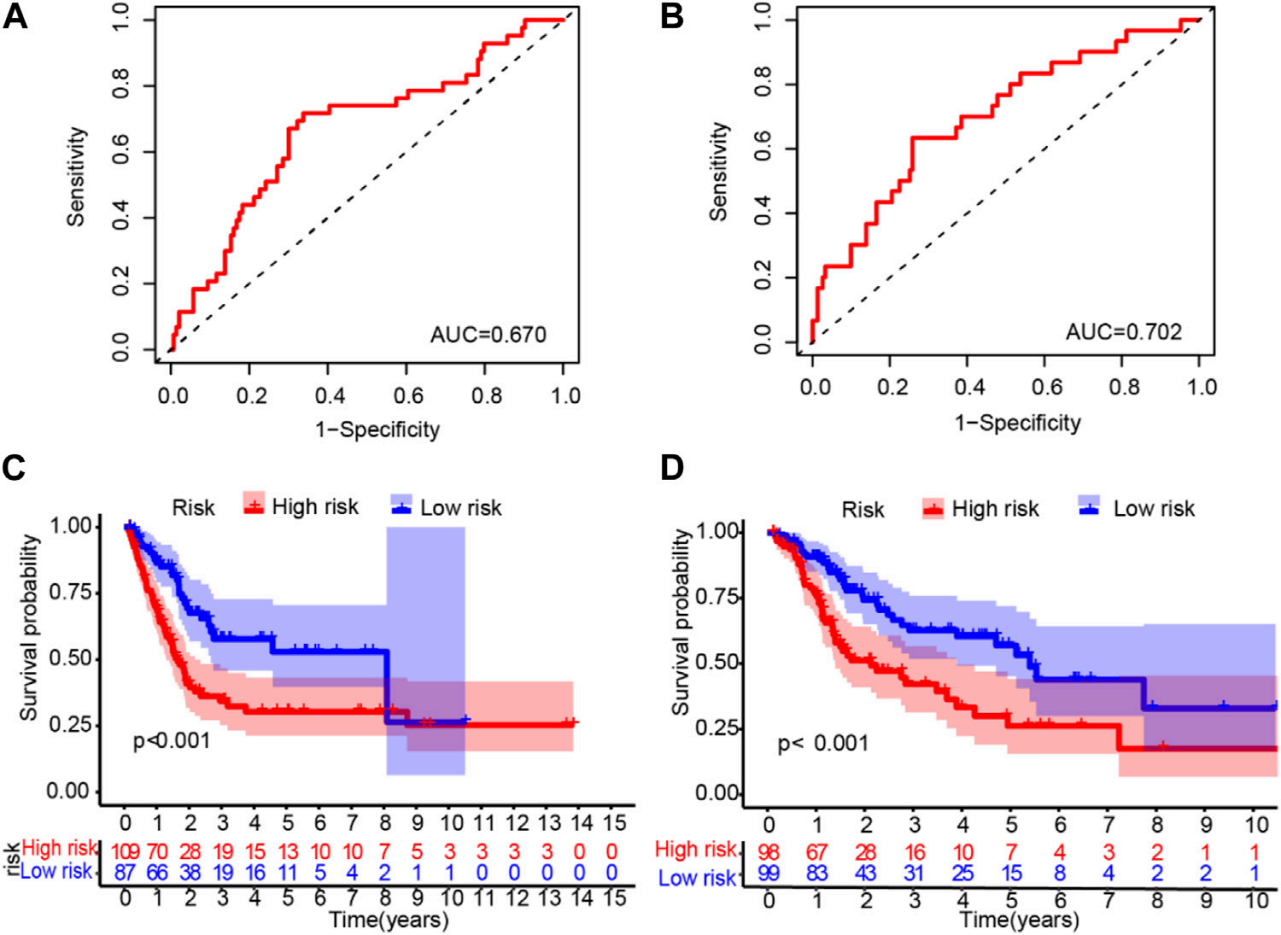
ROC validation and Kaplan‒Meier curve analysis for the lactate-related lncRNA signature. **(A-B)** The ROC areas were 0.670 and 0.702 in validation sets 1 (dataset 2: n = 197) and 2 (dataset 3: 196), respectively. **(C–D)** Prolonged OS in low-risk *versus* high-risk patients in both validation cohorts (log-rank test, *p* < 0.001).

To validate the LM_lncRNA signature, its prognostic accuracy was further evaluated in an independent cohort. These two validation datasets were also downloaded from the TCGA database. Moreover, the data of “Dataset 2” and “Dataset 3” were randomly selected from the 397 patients obtained in the initial part of the present study. Two validation sets were randomly selected: validation set 1 (dataset 2: 197) and validation set 2 (dataset 3: 196). Low-risk patients had significantly longer survival, as evaluated by the ROC curve, with areas of 0.670 and 0.702 (Figures 4A,B) and the validation cohorts (Figures 4C,D), respectively.

### 3.4 Construction of the nomogram in the TCGA cohort

**FIGURE 5:**
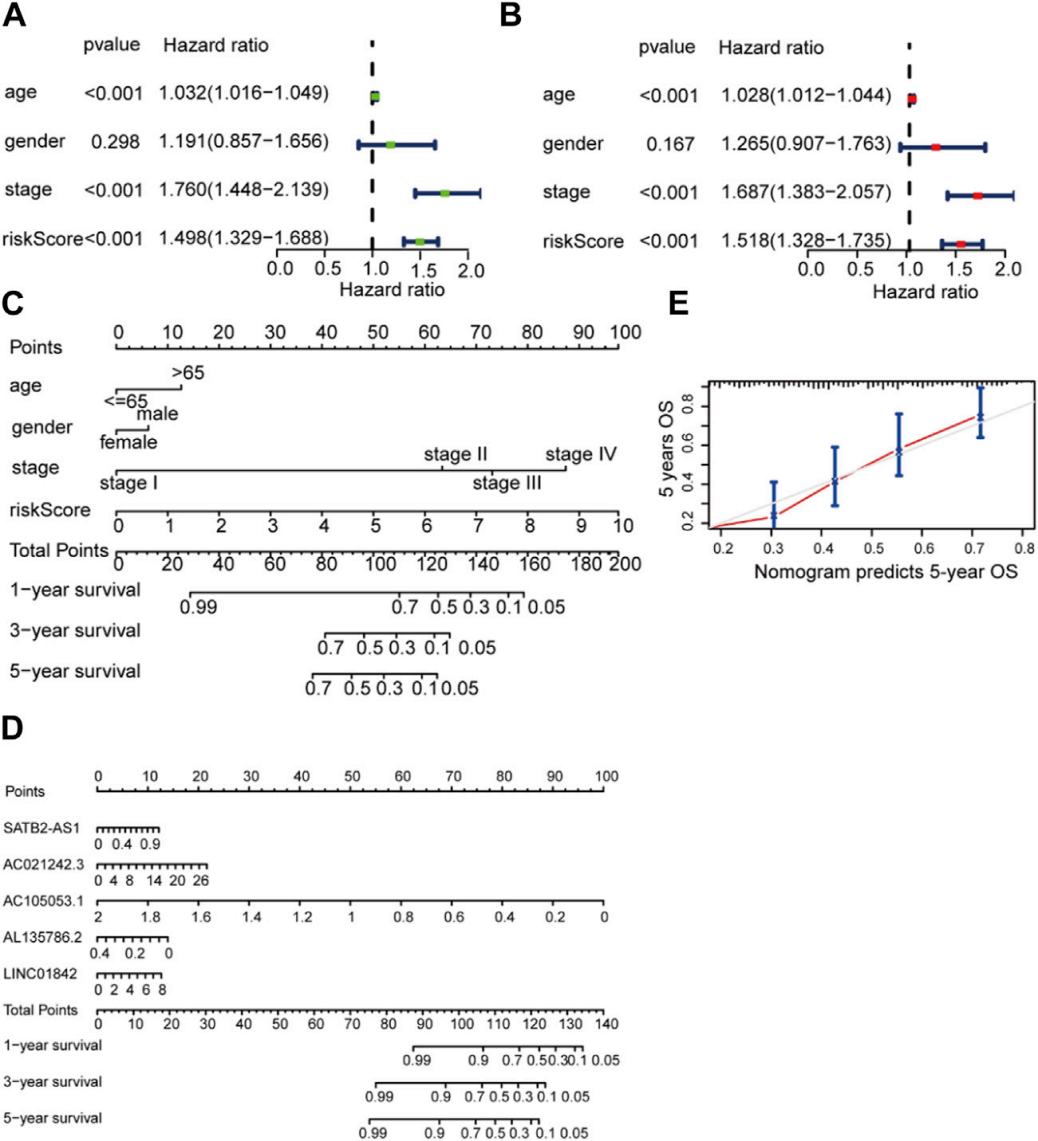
Independent prognostic value of the LDEL risk model. **(A, B)** Univariate **(A)** and multivariate Cox **(B)** analyses in the training cohort. **(C)** A nomogram for the lactate lncRNA signature. **(D)** A nomogram for both prognostic lactate lncRNAs and pathological factors. **(E)** C-index analysis of the nomogram.

According to univariate and multivariate regression analyses, BCA was an independent prognostic factor (Figures 5A,B). Figure 4C shows the nomogram derived from the five LM_lncRNAs. The mixed nomogram (Figure 5D) combining clinicopathological features and prognostic factors of LM_lncRNAs coupled with the 5-year calibration curve could be applied stably and accurately to treat BCA patients (Figure 5E).

**FIGURE 6:**
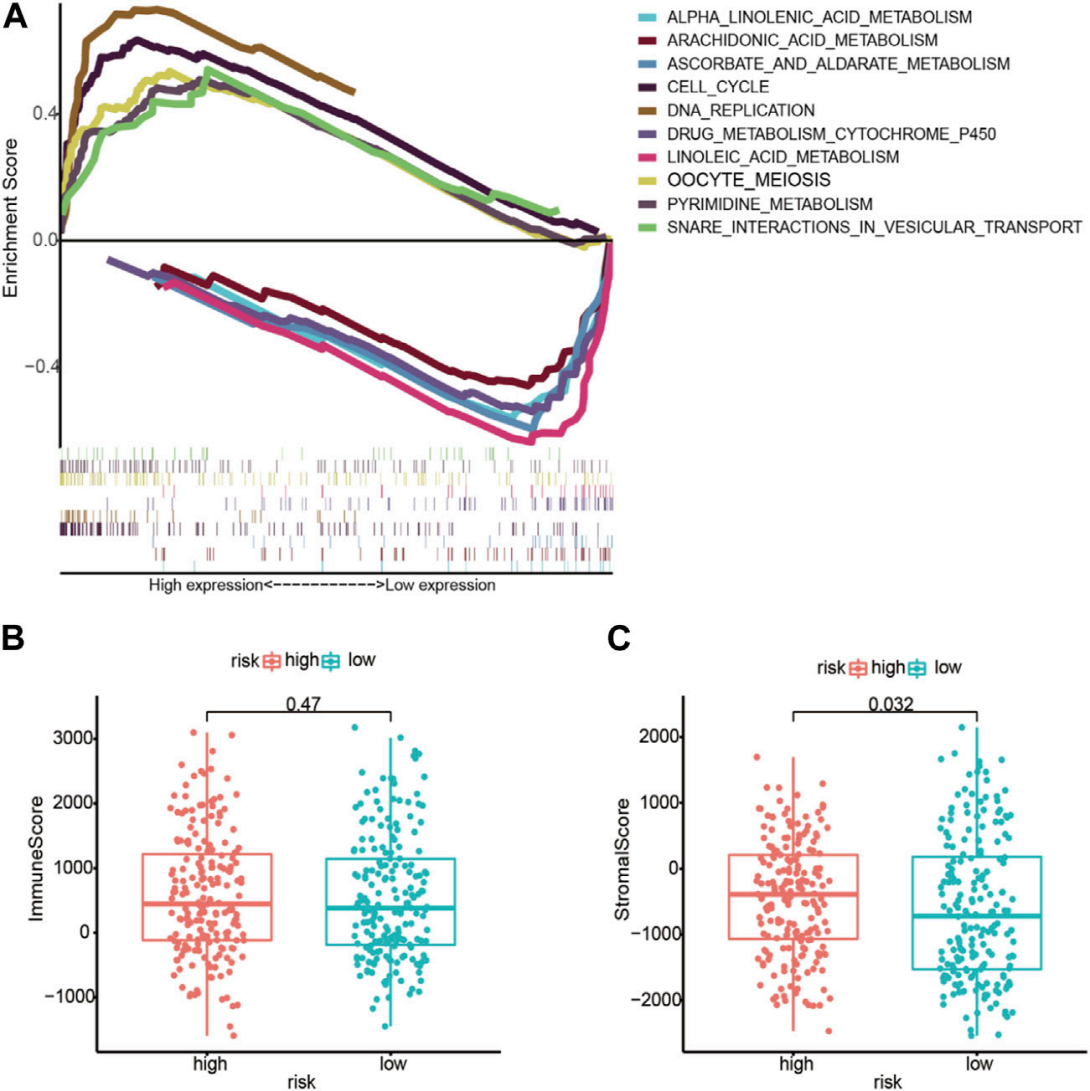
**(A)** Enrichment of genes in the representative pathways by GSEA function analysis. **(B)** Immune scores for the high-risk and low-risk groups. **(C)** The **s**tromal score for the high-risk and low-risk groups.

In low- and high-risk individuals, GSEA identified pathways enriched with differentially expressed lncRNAs. According to these findings, LM_lncRNAs play a central role in cell cycle regulation, oocyte meiosis, pyrimidine metabolism, and DNA replication. Low-risk individuals showed higher steroid hormone biosynthesis, retinol metabolism, and linoleic acid oxidation (Figure 6A).

### 3.5 Subtype-specific genomic profiling and immune infiltration levels

**FIGURE 7:**
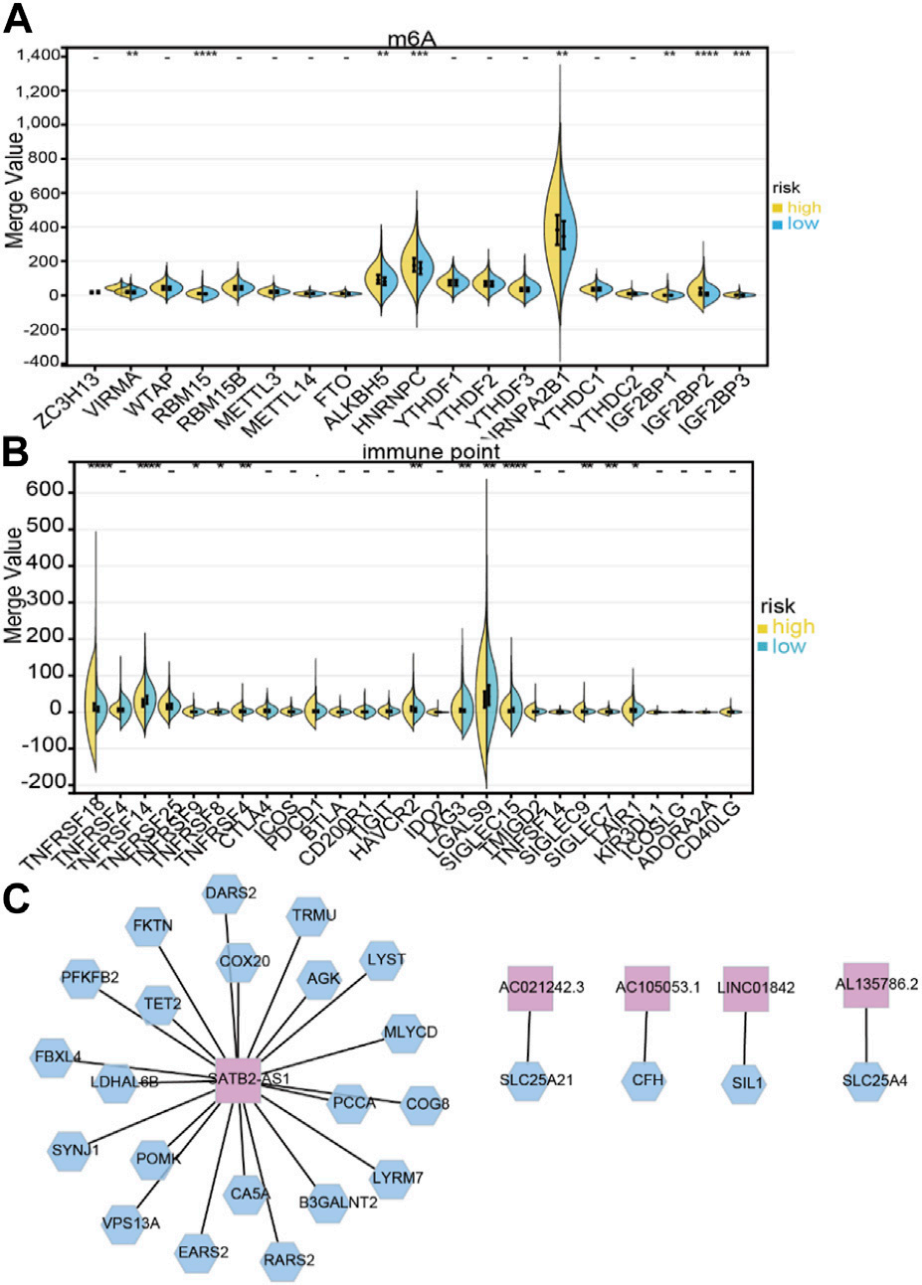
Correlation between LDELs and immunometabolic modification. **(A)** Expression of m^6^A genes between high- and low-risk subgroups (^−^ *p* ≥ 0.1, ·*p* < 0.1, *p < 0.05, **p < 0.01, ***p < 0.001, ****p < 0.0001). **(B)** Distribution of immune checkpoints between the high- and low-risk subgroups. **(C)** Protein‒protein interaction (PPI) network of 5 LDELs and lactate metabolism genes.

Based on immune scores, no significant differences were found between the high- and low-risk groups. In the high-risk group, stromal scores were significantly different from those in the low-risk group; moreover, as shown in Figures 6B,C, immune infiltration of the matrix was significantly different in the high-risk group. As immune checkpoint inhibitors are a critical component of immunotherapy, we explored differences between groups in immune checkpoint expression. HNRNPA2B1, HNRNPC, IGF2BP2, IGF3, ALKBH5, and YTHDF2 were significantly different between the high-risk and low-risk groups in terms of m6A modification (Figure 7A). The two patient groups expressed significantly different levels of lncRNAs, such as TNFRSF18, TNFRSF14, TNFRSF9, TNFRSF8, TNFSF4, HAVCR2, LAG3, LGALS9, SIGLEC15, SIGLEC9, SIGLEC7, and LAIR1 (Figure 7B). According to the results of the Pearson correlation calculation in the previous section, with |*R* ^2^| > 0.3 and *p* < 0.001 as the correlation criteria, to identify independent prognostic factors for LM_mRNAs, a network diagram was drawn (Figure 7C).

### 3.6 Predicting chemotherapeutic response

**FIGURE 8:**
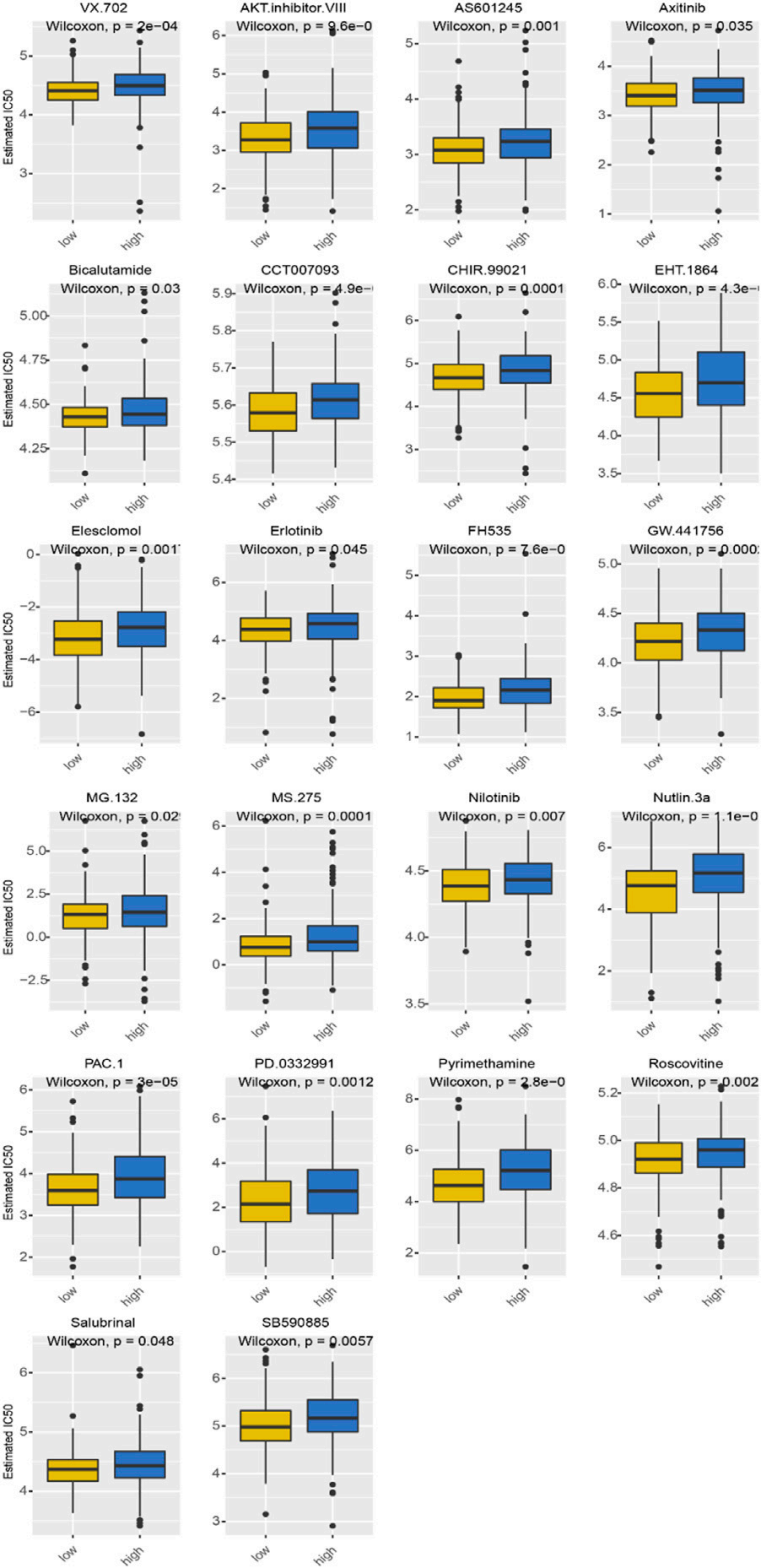
Comparative analysis of chemotherapy drugs with good efficacy in the high-risk group.

We utilized the GDSC website to assess the outcome considering that chemotherapy resistance directly affects patient outcomes. Furthermore, we assessed the response of the two subgroups to chemotherapeutic agents using the GDSC cell line dataset (Figure 8). A total of 22 drugs were found to be more sensitive to high-risk subtypes, increasing a patient’s prognosis when chemotherapy drugs were used on patients with high-risk subtypes Table 1. The above results can help to screen for more suitable chemotherapy drugs for precise treatment. Several specific targeted therapeutic drugs with the smallest IC50, such as AKT inhibitor VIII, AS601245, axitinib, FH535, MG.132, MS.275, and PD.0332991, have more significant potential to be developed into a high-risk group for the treatment of BCA.

## 4 Discussion

BCA has a poor prognosis partly because of the lack of an effective early diagnosis. The clinical diagnosis of BCA mainly relies on cystoscopy biopsy and urine cytology. Cystoscopic biopsy is invasive and expensive, and urine cytology is less sensitive for identifying early low-grade BCA (Lokeshwar et al., 2005). Moreover, due to the lack of sensitivity and specificity of diagnosis, a series of BCA-related biomarkers (such as nuclear matrix protein, bladder tumor antigen, and cytokeratin) have limited application value in the early detection of BCA (Chao et al., 2001). Therefore, developing new biomarkers with high sensitivity and specificity is critical for the early diagnosis and prognostic analysis of UBCA.

The study of this type of UBCA, its molecular mechanism, and the prognosis of MIBCA patients is essential for the prognosis of this type of UBCA. This study first used TCGA and MSigDB data to confirm the LM_mRNAs further screened by correlation analysis. Recent reports indicated that patient data in TCGA with follow-up times <30 days or OS < 30 days were excluded (Dai et al., 2021; Fang et al., 2021; Li et al., 2021). We performed the analysis in the present study according to such a modality.To identify differentially expressed lncRNAs, we conducted a differential analysis on the lncRNAs above. Univariate and multivariate analyses identified LM_lncRNAs that might be independent risk factors for UBCA. This study screened five differentially expressed lncRNAs: SATB2-AS1, AC021242.3, LINC01842, AC105053.1, and AL135786.2. SATB2-AS1, an inhibitor of microRNA155-3p, regulates the migration and proliferation of breast cancer cells (Liu et al., 2017). Studies of SATB2-AS1 in colon tumors demonstrated that it could regulate SATB2 to affect the colon tumor microenvironment (Xu et al., 2019). The lncRNA SATB2-AS1 regulates the proliferation of lung cancer cells by coordinating with other lncRNAs (Lu et al., 2021). In previous studies on lung cancer-related lncRNAs, LINC01842 was considered to regulate lung cancer cell proliferation in a ceRNA pattern with CASC8 and VPS9D1-AS1 (Dai et al., 2020). However, there are no reports on the expression and function of AC105053.1, AC021242.3, and AL135786.2 in tumors. Research on tumor and immune regulation and the TME has gradually become a hotspot in recent years. However, most of the research on the immune regulation-related mechanism of UBCA is limited to animal experiments and direct sequencing data. There needs to be in-depth research on the mechanism, especially the mechanism of lactic acid in UBCA (Conde et al., 2015). The expression levels of immune checkpoints are predictive biomarkers of immunotherapy response, showing broad potential for precision therapeutics. In metastatic UBCA, immunotherapy targeting suppressive immune checkpoints has often been used as a second-line therapy, but only 30% of patients respond to ICI immunotherapy (Lopez-Beltran et al., 2021). Earlier studies related to immunotherapy and pan-cancer research demonstrated that methylation played a critical role in immune cell infiltration (Guo et al., 2021). The process of m6A modification was proven to be the key to methylation (Ma et al., 2019). ALKBH5 regulates target gene splicing, leading to changes in lactate in the tumor microenvironment (Li et al., 2020). METTL3-mediated RNA m6A modification regulates lactate metabolism in the TME (Xiong. et al., 2022). We hypothesize that those with high lactate risk scores may benefit more from immunotherapy. In comparison to low-risk groups, high-risk groups exhibited significantly elevated levels of m6A modification, as well as TNFRSF18, HAVCR2, and LAG3, suggesting that these m6A modification suppressive agents may be considered for patients with a high lactate risk.

There were 22 drugs identified in the GDSC cell line dataset that were highly specific to the high-risk lactate group, which provides new targets for treating UBCA more precisely. By controlling aerobic glycolysis, overactivated PTEN/PI3K/Akt/mTOR promotes cancer metabolic conversion and tumor cell proliferation. AKT inhibitor VIII has been proven to protect gastric cancer cells, clear cell renal cell carcinoma, and breast cancer cells. AS601245, an anti-inflammatory JNK inhibitor, and clofibrate induce cell responses and alter gene expression profiles in Caco-2 colon cancer cells (Cerbone et al., 2012). In addition to inhibiting VEGFR1, VEGFR2, and VEGFR3, axitinib inhibits platelet-derived growth factor receptors and C-Kit. Treatment has been used for advanced renal cell carcinoma patients who have not responded to cytokines or tyrosine inhibitors.

Nevertheless, it is not used in the treatment of BCA. *In vitro* and *in vivo*, blocking the SDF-1/CXCR4/β-catenin axis inhibits the growth of BCA cells, but there are few related reports (Zhang et al., 2018). FH535, an inhibitor of the β-catenin pathway, inhibits the release of the proangiogenic cytokines vascular endothelial growth factor (VEGF), interleukin (IL)-6, IL-8, and TNF-α. It inhibits angiogenesis *in vitro* and *in vivo* (Liu et al., 2016). The proteasome inhibitor MG-132 inhibits mitochondrial-mediated intrinsic myocardial apoptosis and NF-κB-mediated inflammation, and less research has been done on cancer treatment. An investigation of MS-275, a potent cytotoxic HDACi selective for classes I/IV, in RMS xenograft models demonstrated modest antitumor activity alone and combined with standard chemotherapy (Cassandri et al., 2021). A selective CDK4/6 inhibitor, palbociclib, has shown outstanding results in phase II clinical trials in patients with estrogen receptor-positive HER2-negative breast cancer (Bollard et al., 2017).

## 5 Conclusion

Based on ROC analysis, DCA, and calibration curve analysis of the TCGA dataset, we identified a novel, efficient, and highly prognostic LM_lncRNA signature. LM_lncRNAs were found to act as independent predictors of OS in the TCGA database. Validation by random grouping within the dataset shows its effectiveness. In addition, 22 chemotherapeutic agents sensitive to the high-risk group were predicted, which could be used to treat tumors with tumor-related sensitive drugs. This study developed a new method for diagnosing and evaluating UBCA patients’ survival prognoses based on lactate metabolism.

## Data Availability

The original contributions presented in the study are included in the article/Supplementary Material, further inquiries can be directed to the corresponding authors.
