# Supplementary material for: Identification of novel lactate metabolism-related lncRNAs with prognostic value for bladder cancer
Source: Front Pharmacol. 2023 Sep 13;14:1215296. doi: 10.3389/fphar.2023.1215296 (PMC10533998; doi:10.3389/fphar.2023.1215296)
Supplement: Supplementary file 5 [file Table1.DOC]

**Table 1**

| Table 1 The clinical characteristics of patients in the TCGA dataset | |
| --- | --- |
| Variable | Number of samples |
| Gender |  |
| Male/Female | 294/103 |
| Age |  |
| ≤65/>65 | 159/238 |
| Stage |  |
| I/II/III/IV/NA | 2/124/137/132/2 |
| Grade |  |
| High/Low/UN | 376/18/3 |
| T |  |
| T0/T1/T2/T3/T4/UN | 1/3/114/190/58/31 |
| M |  |
| M0/M1/MX/UN | 187/10/198/2 |
| N |  |
| N0/N1/N2/N3/NX/UN | 229/45/76/7/36/4 |
