# Supplementary material for: Identification of novel lactate metabolism-related lncRNAs with prognostic value for bladder cancer
Source: Front Pharmacol. 2023 Sep 13;14:1215296. doi: 10.3389/fphar.2023.1215296 (PMC10533998; doi:10.3389/fphar.2023.1215296)
Supplement: Supplementary file 6 [file Table2.DOCX]

**Trans gene id**

setwd("D:\\bladder\\05.TPMtrans")

rt=read.table("2.TCGA-LIHC-lncRNA.csv",sep=",",header=T,check.names=F)

rt=as.matrix(rt)

rt[1:4,1:4]

rownames(rt)=rt[,1]

exp=rt[,2:ncol(rt)]

dimnames=list(rownames(exp),colnames(exp))

data=matrix(as.numeric(as.matrix(exp)),nrow=nrow(exp),dimnames=dimnames)

library("limma")

data=avereps(data)

data=data[rowMeans(data)>0,]

expMatrix<-data

countToTpm <- function(counts, effLen)

{

rate <- log(counts) - log(effLen)

denom <- log(sum(exp(rate)))

exp(rate - denom + log(1e6))

}

countToFpkm <- function(counts, effLen)

{

N <- sum(counts)

exp( log(counts) + log(1e9) - log(effLen) - log(N) )

}

fpkmToTpm <- function(fpkm)

{

exp(log(fpkm) - log(sum(fpkm)) + log(1e6))

}

tpms <- apply(expMatrix,2,fpkmToTpm)

tpms[1:3,]

colSums(tpms)

write.table(tpms,file="tpm-lncRNA.txt",sep="\t",quote=F,row.names=T,col.names=T)

**pearson**

rm(list = ls())

setwd("D:\\bladder\\07.perasn")

library(limma)

rt=read.table("merge.csv",sep=",",header=T,check.names=F)

rt=as.matrix(rt)

rownames(rt)=rt[,1]

exp=rt[,2:ncol(rt)]

dimnames=list(rownames(exp),colnames(exp))

data=matrix(as.numeric(as.matrix(exp)),nrow=nrow(exp),dimnames=dimnames)

data=avereps(data)

deLNC <- read.table("lncRNA-6380.txt",sep="\t",header=F,check.names=F)

deLNC=c(deLNC[,1])

dePC <- read.table("mRNA-306.txt",sep="\t",header=F,check.names=F)

dePC=c(dePC[,1])

combination <- expand.grid(deLNC, dePC)

names(combination)=c("lnc","pc")

cor_result=apply(combination,1,function(x){

lnc=as.character(x[1])

pc=as.character(x[2])

result=cor.test(as.numeric(data[lnc,]), as.numeric(data[pc,]))

score=c(pval=result$p.value,result$estimate)

return(score)

})

result=cbind(combination,t(cor_result))

head(result)

want = result[(result$pval<0.05 & result$cor>0.3),]

write.table(want, file="want.xls",sep="\t",quote=F)

**different expression**

library("limma")

setwd("D:\\bladder\\08.diff")

inputFile="lactate-lnc-4599Matrix.txt"

fdrFilter=0.05

logFCfilter=1

conNum=19

treatNum=411

outTab=data.frame()

grade=c(rep(1,conNum),rep(2,treatNum))

rt=read.table(inputFile,sep="\t",header=T,check.names=F)

rt=as.matrix(rt)

rownames(rt)=rt[,1]

exp=rt[,2:ncol(rt)]

dimnames=list(rownames(exp),colnames(exp))

data=matrix(as.numeric(as.matrix(exp)),nrow=nrow(exp),dimnames=dimnames)

data=avereps(data)

data=data[rowMeans(data)>0,]

for(i in row.names(data)){

geneName=unlist(strsplit(i,"\\|",))[1]

geneName=gsub("\\/", "_", geneName)

rt=rbind(expression=data[i,],grade=grade)

rt=as.matrix(t(rt))

wilcoxTest<-wilcox.test(expression ~ grade, data=rt)

conGeneMeans=mean(data[i,1:conNum])

treatGeneMeans=mean(data[i,(conNum+1):ncol(data)])

logFC=log2(treatGeneMeans)-log2(conGeneMeans)

pvalue=wilcoxTest$p.value

conMed=median(data[i,1:conNum])

treatMed=median(data[i,(conNum+1):ncol(data)])

diffMed=treatMed-conMed

if( ((logFC>0) & (diffMed>0)) | ((logFC<0) & (diffMed<0)) ){

outTab=rbind(outTab,cbind(gene=i,conMean=conGeneMeans,treatMean=treatGeneMeans,logFC=logFC,pValue=pvalue))

}

}

pValue=outTab[,"pValue"]

fdr=p.adjust(as.numeric(as.vector(pValue)),method="fdr")

outTab=cbind(outTab,fdr=fdr)

write.table(outTab,file="all-lncRNA.txt",sep="\t",row.names=F,quote=F)

outDiff=outTab[( abs(as.numeric(as.vector(outTab$logFC)))>logFCfilter & as.numeric(as.vector(outTab$fdr))<fdrFilter),]

write.table(outDiff,file="diff-lncRNA.txt",sep="\t",row.names=F,quote=F)

heatmap=rbind(ID=colnames(data[as.vector(outDiff[,1]),]),data[as.vector(outDiff[,1]),])

write.table(heatmap,file="diffGeneExp-lncRNA.txt",sep="\t",col.names=F,quote=F)

pdf(file="vol.pdf",height=5,width=5)

xMax=8

outTab[logFC>8]=8

#xMax=max(abs(as.numeric(as.vector(outTab$logFC))))

yMax=max(-log10(outTab$fdr))+1

plot(as.numeric(as.vector(outTab$logFC)), -log10(outTab$fdr), xlab="logFC",ylab="-log10(fdr)",

main="Volcano", ylim=c(0,yMax),xlim=c(-xMax,xMax),yaxs="i",pch=20, cex=0.8)

diffSub=subset(outTab, fdr<fdrFilter & as.numeric(as.vector(logFC))>logFCfilter)

points(as.numeric(as.vector(diffSub$logFC)), -log10(diffSub$fdr), pch=20, col="red",cex=0.8)

diffSub=subset(outTab, fdr<fdrFilter & as.numeric(as.vector(logFC))<(-logFCfilter))

points(as.numeric(as.vector(diffSub$logFC)), -log10(diffSub$fdr), pch=20, col="green",cex=0.8)

abline(v=0,lty=2,lwd=3)

dev.off()

library(pheatmap)

hmExp=data[as.vector(outDiff[,1]),]

hmExp=log2(hmExp+0.01)

Type=c(rep("N",conNum),rep("T",treatNum))

names(Type)=colnames(data)

Type=as.data.frame(Type)

hmExp[hmExp>5]=5

hmExp[hmExp<-5]=-5

pdf(file="heatmap1.pdf",height=6,width=10)

pheatmap(hmExp,

annotation=Type,

color = colorRampPalette(c("green", "black", "red"))(50),

cluster_cols =F,

show_colnames = F,

show_rownames = F,

fontsize = 12,

fontsize_row=10,

fontsize_col=10)

dev.off()

**merge time**

library(limma)

expFile="diffGeneExp-lncRNA.txt"

cliFile="time-397.txt"

setwd("D:\\bladder\\09.mergeTime")

rt=read.table(expFile,sep="\t",header=T,check.names=F)

rt=as.matrix(rt)

rownames(rt)=rt[,1]

exp=rt[,2:ncol(rt)]

dimnames=list(rownames(exp),colnames(exp))

data=matrix(as.numeric(as.matrix(exp)),nrow=nrow(exp),dimnames=dimnames)

data=avereps(data)

data=data[rowMeans(data)>0,]

group=sapply(strsplit(colnames(data),"\\-"),"[",4)

group=sapply(strsplit(group,""),"[",1)

group=gsub("2","1",group)

data=data[,group==0]

colnames(data)=gsub("(.*?)\\-(.*?)\\-(.*?)\\-(.*?)\\-.*","\\1\\-\\2\\-\\3",colnames(data))

data=t(data)

data=avereps(data)

cli=read.table(cliFile,sep="\t",check.names=F,header=T,row.names=1)

sameSample=intersect(row.names(data),row.names(cli))

data=data[sameSample,]

cli=cli[sameSample,]

out=cbind(cli,data)

out=cbind(id=row.names(out),out)

write.table(out,file="expTime.txt",sep="\t",row.names=F,quote=F)

**uniCOX**

library(survival)

setwd("D:\\bladder\\11.uniCox")

pFilter=0.05

rt=read.table("expTime-393.txt",header=T,sep="\t",check.names=F,row.names=1)

rt$futime=rt$futime/365

#rt[,3:ncol(rt)]=log2(rt[,3:ncol(rt)]+1)

diffRT=read.table("diff-lncRNA.txt",header=T,sep="\t",check.names=F,row.names=1)

outTab=data.frame()

sigGenes=c("futime","fustat")

for(gene in colnames(rt[,3:ncol(rt)])){

if(sd(rt[,gene])<0.01){next}

cox=coxph(Surv(futime, fustat) ~ rt[,gene], data = rt)

coxSummary = summary(cox)

coxP=coxSummary$coefficients[,"Pr(>|z|)"]

if( ((coxSummary$conf.int[,"exp(coef)"]>1) & (diffRT[gene,"logFC"]>0)) | ((coxSummary$conf.int[,"exp(coef)"]<1) & (diffRT[gene,"logFC"]<0)) ){

if(coxP<pFilter){

group=ifelse(rt[,gene]>median(rt[,gene]),"high","low")

diff=survdiff(Surv(futime, fustat) ~group,data = rt)

pValue=1-pchisq(diff$chisq,df=1)

if(pValue<pFilter){

sigGenes=c(sigGenes,gene)

outTab=rbind(outTab,

cbind(gene=gene,

#KM=pValue,

HR=coxSummary$conf.int[,"exp(coef)"],

HR.95L=coxSummary$conf.int[,"lower .95"],

HR.95H=coxSummary$conf.int[,"upper .95"],

coxPvalue=coxP) )

}

}

}

}

write.table(outTab,file="uniCox.txt",sep="\t",row.names=F,quote=F)

surSigExp=rt[,sigGenes]

surSigExp=cbind(id=row.names(surSigExp),surSigExp)

write.table(surSigExp,file="uniSigExp.txt",sep="\t",row.names=F,quote=F)

**MultiCOX**

library(survival)

setwd("D:\\bladder\\12.multiCox")

rt=read.table("uniSigExp.txt",header=T,sep="\t",check.names=F,row.names=1)

multiCox=coxph(Surv(futime, fustat) ~ ., data = rt)

multiCox=step(multiCox,direction = "both")

multiCoxSum=summary(multiCox)

outTab=data.frame()

outTab=cbind(

coef=multiCoxSum$coefficients[,"coef"],

HR=multiCoxSum$conf.int[,"exp(coef)"])

outTab=cbind(id=row.names(outTab),outTab)

outTab=gsub("`","",outTab)

write.table(outTab,file="multiCox.xls",sep="\t",row.names=F,quote=F)

riskScore=predict(multiCox,type="risk",newdata=rt)

coxGene=rownames(multiCoxSum$coefficients)

coxGene=gsub("`","",coxGene)

outCol=c("futime","fustat",coxGene)

risk=as.vector(ifelse(riskScore>median(riskScore),"high","low"))

write.table(cbind(id=rownames(cbind(rt[,outCol],riskScore,risk)),cbind(rt[,outCol],riskScore,risk)),

file="risk.txt",

sep="\t",

quote=F,

row.names=F)

**Risk survival**

library(survival)

setwd("D:\\bladder\\13.riskSurvival")

data=read.table("risk.txt",header=T,sep="\t",check.names=F)

diff=survdiff(Surv(futime, fustat) ~risk,data = data)

pValue=1-pchisq(diff$chisq,df=1)

pValue=signif(pValue,4)

pValue=format(pValue, scientific = TRUE)

fit <- survfit(Surv(futime, fustat) ~ risk, data = data)

pdf(file="survival.pdf",width=5.5,height=5)

plot(fit,

lwd=2,

col=c("red","blue"),

xlab="Time (year)",

ylab="Survival rate",

main=paste("Survival curve (p=", pValue ,")",sep=""),

mark.time=T)

legend("topright",

c("High risk", "Low risk"),

lwd=2,

col=c("red","blue"))

dev.off()

summary(fit)

**ROC**

library(survivalROC)

setwd("D:\\bladder\\16.multiROC")

rt=read.table("indepInput-395.txt",header=T,sep="\t",check.names=F,row.names=1)

rocCol=rainbow(ncol(rt)-2)

aucText=c()

rt$futime=rt$futime/365

#????risk score??ROC????

pdf(file="multiROC.pdf",width=6,height=6)

par(oma=c(0.5,1,0,1),font.lab=1.5,font.axis=1.5)

roc=survivalROC(Stime=rt$futime, status=rt$fustat, marker = rt$riskScore, predict.time =5, method="KM")

plot(roc$FP, roc$TP, type="l", xlim=c(0,1), ylim=c(0,1),col=rocCol[1],

xlab="False positive rate", ylab="True positive rate",

lwd = 2, cex.main=1.3, cex.lab=1.2, cex.axis=1.2, font=1.2)

aucText=c(aucText,paste0("risk score"," (AUC=",sprintf("%.3f",roc$AUC),")"))

abline(0,1)

j=1

for(i in colnames(rt[,3:(ncol(rt)-1)])){

roc=survivalROC(Stime=rt$futime, status=rt$fustat, marker = rt[,i], predict.time =5, method="KM")

j=j+1

aucText=c(aucText,paste0(i," (AUC=",sprintf("%.3f",roc$AUC),")"))

lines(roc$FP, roc$TP, type="l", xlim=c(0,1), ylim=c(0,1),col=rocCol[j],lwd = 2)

}

legend("bottomright", aucText,lwd=2,bty="n",col=rocCol)

dev.off()

**C index**

library(survival)

setwd("D:\\bladder\\18.C-index")

inputfile="risk.txt"

lncRNA<-read.table(inputfile,header=T,sep="\t",row.names = 1,check.names = F,stringsAsFactors = F)

lncRNAEXP=lncRNA[,3:ncol(lncRNA)]

lncRNA=cbind(lncRNA[,1:2],lncRNAEXP)

mycox<- coxph(Surv(futime,fustat)~.,data = lncRNA)

summary(mycox)

**estimate：**

library(limma)

library(estimate)

setwd("D:\\bladder\\18.estimate")

inputFile="symbol.txt"

rt=read.table(inputFile,sep="\t",header=T,check.names=F)

rt=as.matrix(rt)

rownames(rt)=rt[,1]

#exp=rt

exp=rt[,2:ncol(rt)]

dimnames=list(rownames(exp),colnames(exp))

data=matrix(as.numeric(as.matrix(exp)),nrow=nrow(exp),dimnames=dimnames)

data=avereps(data)

group=sapply(strsplit(colnames(data),"\\-"),"[",4)

group=sapply(strsplit(group,""),"[",1)

group=gsub("2","1",group)

data=data[,group==0]

out=data[rowMeans(data)>0,]

out=rbind(ID=colnames(out),out)

write.table(out,file="uniq.symbol.txt",sep="\t",quote=F,col.names=F)

filterCommonGenes(input.f="uniq.symbol.txt",

output.f="commonGenes.gct",

id="GeneSymbol")

estimateScore(input.ds = "commonGenes.gct",

output.ds="estimateScore.gct",

platform="illumina")

scores=read.table("estimateScore.gct",skip = 2,header = T)

rownames(scores)=scores[,1]

scores=t(scores[,3:ncol(scores)])

rownames(scores)=gsub("\\.","\\-",rownames(scores))

out=rbind(ID=colnames(scores),scores)

write.table(out,file="scores.txt",sep="\t",quote=F,col.names=F)

**riskplot**

library(pheatmap)

setwd("D:\\bladder\\19.riskPlot")

rt=read.table("risk.txt",sep="\t",header=T,row.names=1,check.names=F)

rt=rt[order(rt$riskScore),]

#???品???????

riskClass=rt[,"risk"]

lowLength=length(riskClass[riskClass=="low"])

highLength=length(riskClass[riskClass=="high"])

line=rt[,"riskScore"]

line[line>10]=10

pdf(file="riskScore.pdf",width = 10,height = 3.5)

plot(line,

type="p",

pch=20,

xlab="Patients (increasing risk socre)",

ylab="Risk score",

col=c(rep("green",lowLength),

rep("red",highLength)))

abline(h=median(rt$riskScore),v=lowLength,lty=2)

legend("topleft", c("High risk", "low Risk"),bty="n",pch=19,col=c("red","green"),cex=1.2)

dev.off()

color=as.vector(rt$fustat)

color[color==1]="red"

color[color==0]="green"

pdf(file="survStat.pdf",width = 10,height = 3.5)

plot(rt$futime,

pch=19,

xlab="Patients (increasing risk socre)",

ylab="Survival time (years)",

col=color)

legend("topleft", c("Dead", "Alive"),bty="n",pch=19,col=c("red","green"),cex=1.2)

abline(v=lowLength,lty=2)

dev.off()

rt1=log2(rt[c(3:(ncol(rt)-2))]+0.01)

rt1=t(rt1)

annotation=data.frame(type=rt[,ncol(rt)])

rownames(annotation)=rownames(rt)

pdf(file="heatmap.pdf",width = 10,height = 3)

pheatmap(rt1,

annotation=annotation,

cluster_cols = FALSE,

fontsize_row=11,

show_colnames = F,

fontsize_col=3,

color = colorRampPalette(c("green", "black", "red"))(50) )

dev.off()

**calibration curve**

library(rms)

lisetwd("D:\\bladder\\17校准????")

rm(list = ls())

aa=read.table("indepInput-395.txt",header=T,sep="\t",check.names=F,row.names=1)

names(aa)

for (i in names(aa)[c(3:5)]){aa[,i] <- as.factor(aa[,i])}

nomo<-datadist(aa)

options(datadist='nomo')

fustat=as.numeric(aa$fustat)

nomo1 <- cph(Surv(futime,fustat==1)~age+riskScore+stage+gender,

x=T,y=T,

data=aa,

surv=T,

time.inc=5

)

p<- calibrate(nomo1,

cmethod='KM',

method='boot',

u=5,

m=73,

B=1000)

plot(p,

add=F,

conf.int=T,#95%CI

subtitles = F

cex.subtitles=0.8,

lwd=2,

lty=1,

errbar.col="blue",

xlim=c(0.2,1),

ylim=c(0.2,1),

xlab=" ",

ylab=" ",

col="red")

----------------------------

nomo1 <- cph(Surv(futime,fustat==1)~age+riskScore+stage+gender,

x=T,y=T,

data=aa,

surv=T,

time.inc=3)

p<- calibrate(nomo1,

cmethod='KM',

method='boot',

u=3,

m=73,

B=1000)

plot(p,

add=T,

conf.int=T,

subtitles = F,

cex.subtitles=0.8,

lwd=2,

lty=1,

errbar.col="orange",

xlim=c(0.25,1),

ylim=c(0.25,1),

xlab=" ",

ylab=" ",

col="#407600")

abline(0,1,lty=3,lwd=1,col="grey")

legend("bottomright", legend=c("5???", "3???"), col=c("red", "407600"), lwd=1)

abline(0,1,lty=3,lwd=1,col="grey")

**nomogram**

library(rms)

setwd("D:\\bladder\\21.Nomogram1")

rt=read.table("indepInput-395.txt",sep="\t",header=T,row.names=1,check.names=F)

rt$futime=rt$futime/365

dd <- datadist(rt)

options(datadist="dd")

s1 <- Surv(rt$futime, rt$fustat)

f <- cph(s1 ~ age + gender + stage+ riskScore, x=T, y=T, surv=T, data=rt, time.inc=1)

surv <- Survival(f)

nom <- nomogram(f, fun=list(function(x) surv(1, x), function(x) surv(3, x), function(x) surv(5, x)),

lp=F, funlabel=c("1-year survival", "3-year survival", "5-year survival"),

maxscale=100,

fun.at=c(0.99, 0.7, 0.5, 0.3,0.1,0.05))

pdf(file="nomogram.pdf",height=6,width=10)

plot(nom)

dev.off()

DCA

library(rmda)

setwd('D:\\bladder\\22.DCA')

Data<- read.table("indepInput-395.txt",header=T,sep="\t",row.names = 1)

simple_1<- decision_curve(status~age,data = Data, family = binomial(link ='logit'),

thresholds= seq(0,1, by = 0.01) ,

confidence.intervals =0.95 , study.design = 'cohort')

simple_2<- decision_curve(status~gender,data = Data, family = binomial(link ='logit'),

thresholds= seq(0,1, by = 0.01) ,

confidence.intervals =0.95 , study.design = 'cohort')

simple_3<- decision_curve(status~stage,data = Data, family = binomial(link ='logit'),

thresholds= seq(0,1, by = 0.01) ,

confidence.intervals =0.95 , study.design = 'cohort')

simple_4<- decision_curve(status~pT,data = Data, family = binomial(link ='logit'),

thresholds= seq(0,1, by = 0.01) ,

confidence.intervals =0.95 , study.design = 'cohort')

simple_5<- decision_curve(status~pN,data = Data, family = binomial(link ='logit'),

thresholds= seq(0,1, by = 0.01) ,

confidence.intervals =0.95 , study.design = 'cohort')

simple_6<- decision_curve(status~pM,data = Data, family = binomial(link ='logit'),

thresholds= seq(0,1, by = 0.01) ,

confidence.intervals =0.95 , study.design = 'cohort')

simple_7<- decision_curve(status~riskScore,data = Data, family = binomial(link ='logit'),

thresholds= seq(0,1, by = 0.01) ,

confidence.intervals =0.95 , study.design = 'cohort')

List<- list(simple_1,simple_2,simple_3,simple_7)

plot_decision_curve(List,curve.names= c('Age model','Gender model','Stage model','RiskScore model'),

cost.benefit.axis =T,cex=0.4,

n.cost.benefits= 6 , col = c("red","blue","black","orange"),

confidence.intervals =FALSE,standardize = FALSE)

legend("topright",

legend=c('Age model','Gender model','Stage model','RiskScore model'),

col=c("red","blue","black","orange"),lwd=1, xpd = T, xjust = 0, yjust = -0.28)

**ggalluvial**

library(ggalluvial)

library(ggplot2)

library(dplyr)

setwd("D:\\biowolf\\ARGlncRNA\\15.ggalluvial")

rt=read.table("network.txt",sep = "\t",header = T)

cox=read.table("multiCox.txt",sep="\t",header=T,row.names=1)

protectGene=row.names(cox[cox$HR<1,])

riskType=ifelse(rt$lncRNA%in%protectGene,"Protect","Risk")

newData=cbind(rt[,c(1,2)],riskType)

corLodes=to_lodes_form(newData, axes = 1:3, id = "Cohort")

pdf(file="ggalluvial.pdf",width=7,height=6)

mycol <- rep(c("#029149","#6E568C","#E0367A","#D8D155","#223D6C","#D20A13","#431A3D","#91612D","#FFD121","#088247","#11AA4D","#58CDD9","#7A142C","#5D90BA","#64495D","#7CC767"),5)

ggplot(corLodes, aes(x = x, stratum = stratum, alluvium = Cohort,fill = stratum, label = stratum)) +

scale_x_discrete(expand = c(0, 0)) +

geom_flow(width = 1/8,aes.flow = "forward") +

geom_stratum(alpha = .9,width = 1/10) +

scale_fill_manual(values = mycol) +

geom_text(stat = "stratum", size = 2.4,color="black") +

xlab("") + ylab("") + theme_bw() +

theme(axis.line = element_blank(),axis.ticks = element_blank(),axis.text.y = element_blank()) +

theme(panel.grid =element_blank()) +

theme(panel.border = element_blank()) +

ggtitle("") + guides(fill = FALSE)

dev.off()

**model**

library(survival)

library(caret)

library(glmnet)

library(survminer)

library(timeROC)

coxPfilter=0.05

setwd("D:\\bladder\\temp\\22.model")

rt=read.table("expTime-393.txt",header=T,sep="\t",check.names=F,row.names=1)

rt$futime=rt$futime/365

bioForest=function(coxFile=null,forestFile=null,forestCol=null){

rt <- read.table(coxFile,header=T,sep="\t",row.names=1,check.names=F)

gene <- rownames(rt)

hr <- sprintf("%.3f",rt$"HR")

hrLow <- sprintf("%.3f",rt$"HR.95L")

hrHigh <- sprintf("%.3f",rt$"HR.95H")

Hazard.ratio <- paste0(hr,"(",hrLow,"-",hrHigh,")")

pVal <- ifelse(rt$pvalue<0.001, "<0.001", sprintf("%.3f", rt$pvalue))

pdf(file=forestFile, width = 7, height = 4.5)

n <- nrow(rt)

nRow <- n+1

ylim <- c(1,nRow)

layout(matrix(c(1,2),nc=2),width=c(3,2.5))

xlim = c(0,3)

par(mar=c(4,2.5,2,1))

plot(1,xlim=xlim,ylim=ylim,type="n",axes=F,xlab="",ylab="")

text.cex=0.8

text(0,n:1,gene,adj=0,cex=text.cex)

text(1.5-0.5*0.2,n:1,pVal,adj=1,cex=text.cex);text(1.5-0.5*0.2,n+1,'pvalue',cex=text.cex,adj=1)

text(3,n:1,Hazard.ratio,adj=1,cex=text.cex);text(3,n+1,'Hazard ratio',cex=text.cex,adj=1,)

par(mar=c(4,1,2,1),mgp=c(2,0.5,0))

xlim = c(0,max(as.numeric(hrLow),as.numeric(hrHigh)))

plot(1,xlim=xlim,ylim=ylim,type="n",axes=F,ylab="",xaxs="i",xlab="Hazard ratio")

arrows(as.numeric(hrLow),n:1,as.numeric(hrHigh),n:1,angle=90,code=3,length=0.05,col="darkblue",lwd=2.5)

abline(v=1,col="black",lty=2,lwd=2)

boxcolor = ifelse(as.numeric(hr) > 1, forestCol[1], forestCol[2])

points(as.numeric(hr), n:1, pch = 15, col = boxcolor, cex=1.3)

axis(1)

dev.off()

}

#对分组进行循环，找出train和test都显著的分组

for(i in 1:1000){

inTrain<-createDataPartition(y=rt[,3], p=0.5, list=F)

train<-rt[inTrain,]

test<-rt[-inTrain,]

trainOut=cbind(id=row.names(train),train)

testOut=cbind(id=row.names(test),test)

outUniTab=data.frame()

sigGenes=c("futime","fustat")

for(i in colnames(train[,3:ncol(train)])){

#cox分析

cox <- coxph(Surv(futime, fustat) ~ train[,i], data = train)

coxSummary = summary(cox)

coxP=coxSummary$coefficients[,"Pr(>|z|)"]

#KM分析

#group=ifelse(train[,i]>median(train[,i]),"high","low")

#diff=survdiff(Surv(futime, fustat) ~group,data = train)

#pValue=1-pchisq(diff$chisq,df=1)

if(coxP<coxPfilter){

sigGenes=c(sigGenes,i)

outUniTab=rbind(outUniTab,

cbind(id=i,

HR=coxSummary$conf.int[,"exp(coef)"],

HR.95L=coxSummary$conf.int[,"lower .95"],

HR.95H=coxSummary$conf.int[,"upper .95"],

pvalue=coxSummary$coefficients[,"Pr(>|z|)"])

)

}

}

uniSigExp=train[,sigGenes]

uniSigExpOut=cbind(id=row.names(uniSigExp),uniSigExp)

#############构建COX模型#############

multiCox <- coxph(Surv(futime, fustat) ~ ., data = uniSigExp)

multiCox=step(multiCox,direction = "both")

multiCoxSum=summary(multiCox)

outMultiTab=data.frame()

outMultiTab=cbind(

coef=multiCoxSum$coefficients[,"coef"],

HR=multiCoxSum$conf.int[,"exp(coef)"],

HR.95L=multiCoxSum$conf.int[,"lower .95"],

HR.95H=multiCoxSum$conf.int[,"upper .95"],

pvalue=multiCoxSum$coefficients[,"Pr(>|z|)"])

outMultiTab=cbind(id=row.names(outMultiTab),outMultiTab)

riskScore=predict(multiCox,type="risk",newdata=train)

coxGene=rownames(multiCoxSum$coefficients)

coxGene=gsub("`","",coxGene)

outCol=c("futime","fustat",coxGene)

medianTrainRisk=median(riskScore)

risk=as.vector(ifelse(riskScore>medianTrainRisk,"high","low"))

trainRiskOut=cbind(id=rownames(cbind(train[,outCol],riskScore,risk)),cbind(train[,outCol],riskScore,risk))

riskScoreTest=predict(multiCox,type="risk",newdata=test)

riskTest=as.vector(ifelse(riskScoreTest>medianTrainRisk,"high","low"))

testRiskOut=cbind(id=rownames(cbind(test[,outCol],riskScoreTest,riskTest)),cbind(test[,outCol],riskScore=riskScoreTest,risk=riskTest))

diff=survdiff(Surv(futime, fustat) ~risk,data = train)

pValue=1-pchisq(diff$chisq, df=1)

diffTest=survdiff(Surv(futime, fustat) ~riskTest,data = test)

pValueTest=1-pchisq(diffTest$chisq, df=1)

predictTime=1 #预测时间

roc=timeROC(T=train$futime, delta=train$fustat,

marker=riskScore, cause=1,

weighting='aalen',

times=c(predictTime), ROC=TRUE)

rocTest=timeROC(T=test$futime, delta=test$fustat,

marker=riskScoreTest, cause=1,

weighting='aalen',

times=c(predictTime), ROC=TRUE)

if((pValue<0.01) & (roc$AUC[2]>0.68) & (pValueTest<0.05) & (rocTest$AUC[2]>0.63)){

write.table(trainOut,file="train.data.txt",sep="\t",quote=F,row.names=F)

write.table(testOut,file="test.data.txt",sep="\t",quote=F,row.names=F)

write.table(outUniTab,file="trainUniCox.txt",sep="\t",row.names=F,quote=F)

write.table(uniSigExpOut,file="tcgaUniSigExp.txt",sep="\t",row.names=F,quote=F)

bioForest(coxFile="trainUniCox.txt",forestFile="trainUniCox.pdf",forestCol=c("red","green"))

write.table(outMultiTab,file="multiCox.txt",sep="\t",row.names=F,quote=F)

write.table(testRiskOut,file="testRisk.txt",sep="\t",quote=F,row.names=F)

write.table(trainRiskOut,file="trainRisk.txt",sep="\t",quote=F,row.names=F)

allRiskOut=rbind(trainRiskOut, testRiskOut)

write.table(allRiskOut,file="allRisk.txt",sep="\t",quote=F,row.names=F)

break

}

}

**Survival**

library(survival)

library(survminer)

setwd("D:\\bladder\\temp\\24.survival") #设置工作目录

bioSurvival=function(inputFile=null,outFile=null){

rt=read.table(inputFile,header=T,sep="\t")

#比较高低风险组生存差异，得到显著性p值

diff=survdiff(Surv(futime, fustat) ~risk,data = rt)

pValue=1-pchisq(diff$chisq,df=1)

if(pValue<0.001){

pValue="p<0.001"

}else{

pValue=paste0("p=",sprintf("%.03f",pValue))

}

fit <- survfit(Surv(futime, fustat) ~ risk, data = rt)

surPlot=ggsurvplot(fit,

data=rt,

conf.int=T,

pval=pValue,

pval.size=6,

legend.title="Risk",

legend.labs=c("High risk", "Low risk"),

xlab="Time(years)",

break.time.by = 1,

palette=c("red", "blue"),

risk.table=TRUE,

risk.table.title="",

risk.table.col = "strata",

risk.table.height=.25)

pdf(file=outFile,onefile = FALSE,width = 6.5,height =5.5)

print(surPlot)

dev.off()

}

bioSurvival(inputFile="trainRisk.txt",outFile="trainSurv.pdf")

bioSurvival(inputFile="testRisk.txt",outFile="testSurv.pdf")

bioSurvival(inputFile="allRisk.txt",outFile="allSurv.pdf")
